# Protozoan Parasites in Adult Dairy Small Ruminants and Potential Predictors for Their Presence in Faecal Samples

**DOI:** 10.3390/microorganisms10101931

**Published:** 2022-09-28

**Authors:** Daphne T. Lianou, Konstantinos V. Arsenopoulos, Charalambia K. Michael, Elias Papadopoulos, George C. Fthenakis

**Affiliations:** 1 Veterinary Faculty, University of Thessaly, 43100 Karditsa, Greece; 2 Laboratory of Parasitology and Parasitic Diseases, Faculty of Health Sciences, School of Veterinary Medicine, Aristotle University of Thessaloniki, 54124 Thessaloniki, Greece

**Keywords:** *Cryptosporidium*, diarrhoeic syndrome, *Eimeria*, *Giardia*, lamb, goat, goat kid, predictor, sheep

## Abstract

There is a scope to study protozoan infections in adult ewes and does, as these animals can act as reservoirs of infection for lambs and kids, for which these pathogens are harmful. The objectives of this study were to describe the prevalence of protozoan infections in faecal samples from adult sheep and goats on dairy farms across Greece and to evaluate farm-related factors potentially associated with the presence of protozoan infections in these animals. A cross-sectional study was performed on 325 sheep and 119 goat farms throughout Greece; faecal samples were collected from ewes and does and processed for the identification of protozoan parasites. *Eimeria* oocysts were found in faecal samples from 69% of farms (72% of sheep farms and 61% of goat farms), *Giardia* cysts in samples from 33% of farms (33% of sheep farms and 34% of goat farms) and *Cryptosporidium* oocysts in samples from 8% of farms (7% of sheep farms and 11% of goat farms). In a multivariable analysis, for the presence of *Eimeria* in samples from sheep farms, the lack of a designated building for lambs emerged as a significant factor; for the presence of *Giardia* in samples from goat farms, the availability of a main building for animals emerged as a significant factor; for the presence of *Cryptosporidium*, the lack of grazing and the management system emerged as the main significant factors in sheep and goat farms, respectively. Protozoa were found significantly more frequently in samples collected from farms on which farmers considered diarrhoea as an important health problem in their lambs/kids.

## 1. Introduction

On small ruminant farms, diarrhoeic syndrome in lambs and kids is a significant cause of morbidity and mortality. The causal agents of the syndrome include various pathogens, among which the protozoan parasites *Eimeria*, *Cryptosporidium*, and *Giardia* play a particularly significant role [1]. The reservoirs for these parasites on the farms are the adult animals, in which these protozoa cause few, if any, clinical effects. Hence, parasitised adult animals harbour the protozoa and excrete oocysts or cysts, which lead to infection in lambs or kids [2]. Young animals acquire the infection by ingesting infective forms whilst sucking their dams or after licking the infected, contaminated floor bedding [2].

In general, studies investigating protozoan infections in lambs and kids have been performed primarily on meat production farms. Nevertheless, even on dairy farms, newborns provide important income for farmers, and their diseases cause financial constraints. Previous research related to protozoan infections in lambs and kids have usually targeted infected newborns and have presented findings related to those animals [3,4,5,6,7].

Coccidiosis is the primary protozoan infection in lambs and kids. The main causal agents of coccidiosis are *Eimeria* and *Cryptosporidium*. A variety of the *Eimeria* species can infect lambs and kids, but *Eimeria crandallis* and *E. ovinoidalis* in sheep and *E. ninakohlyakimovae* in goats are considered to be the most pathogenic species [3]. These parasites can cause severe disease in lambs and kids aged 4 to 6 weeks, but in adult animals, the infection remains asymptomatic. *Eimeria* parasites are characterised by host specificity; hence, zoonotic infections by ovine or caprine species are rare [3]. *Cryptosporidium parvum* is the species primarily affecting lambs and kids at the age of one to two weeks. The infection can cause severe disease in young animals, potentially leading to death, but adults undergo non-clinical infections and have a significant role in shedding oocysts of the parasite, particularly around the lambing/kidding period, and thus contribute to the infection of newborns [8]. The parasite has a zoonotic potential, and, in fact, *C. hominis* (previously named type I *C. parvum*) is mostly isolated from people [8]. *Giardia duodenalis* (also named *G. intestinalis* and *G. lamblia*) can infect most mammalian species and comprises a complex of eight different assemblages (often also described as separate species); of these, assemblage E is the one that more often infects livestock species. Animals younger than six months are more susceptible to the infection [9], whilst adults harbour the parasite and contribute to its dissemination within a farm by excreting infective oocysts in their faeces. The parasite has a well-defined zoonotic danger, with assemblages A and B being the ones most often causing infections in people [9]. All three protozoa are transmitted by the faecal–oral route. Thus, some management practices, e.g., a high stocking rate or increased housing period of animals, increase the risk for infection by these protozoa and can contribute to more severe disease in affected animals [1,10].

There is a scope to study protozoan infections in adult ewes and does, as the adult animals act as reservoirs of infection for the lambs and kids. The adults excrete oocysts or cysts of the protozoa, which, during the lambing/kidding season and the initial stages of the milking period, can contribute to the infection of the newborns. The objectives of this study were (a) to describe the prevalence of protozoan infections in faecal samples from adult sheep and goats on dairy farms across Greece and (b) to evaluate farm-related factors potentially associated with the presence of protozoan infections in these animals.

## 2. Materials and Methods

### 2.1. Farms

**Figure 1:**
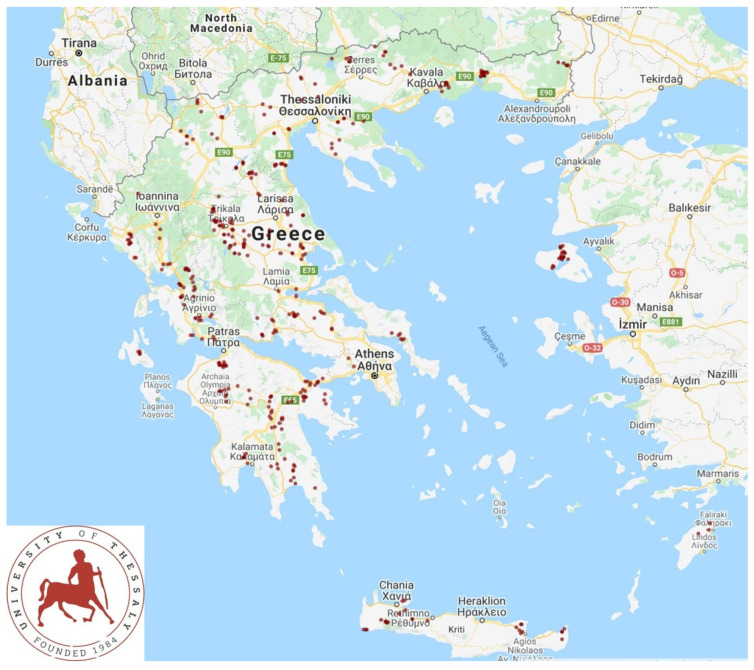
Map of the 444 sheep and goat farms throughout Greece, which were visited for faecal sampling.

A cross-sectional study involving 444 small ruminant farms (325 sheep flocks and 119 goat herds) was performed from April 2019 to July 2020 and covered all the 13 administrative regions of Greece (Figure 1). The procedures and methodology for including farms into the study have been presented, in detail, before [11,12,13]. A structured questionnaire was used to collect information regarding practices applied on the farm, as detailed before [11,12,13]. Antiparasitic administration to adult animals on these farms referred only to the administration of anthelmintic (at standard dose rates, licenced against sheep or goat helminths) and ectoparasiticide drugs, with no use of antiprotozoal drugs.

### 2.2. Collection of Faecal Samples and Examination of Animals

Faecal samples for parasitological examination were collected directly from the rectum of female adult sheep or goats on the farms. On each farm, 20, 30, 40, or 50 ewes or does in the milking period (for farms with ≤165, 166–330, 331–500, or >500 ewes or does, respectively) were selected for sampling. For the selection of subjects for sampling, the animals were walked into the milking area, and the necessary number was selected by using an electronic random number generator (www.randomresult.com).

For the collection of faecal samples, each animal sampled was restrained appropriately. The person collecting the faecal sample was wearing disposable clean latex gloves. One drop of lubricant was applied on the gloved index and middle fingers, which were subsequently inserted into the rectum, taking all due care to avoid potential injury to the animal. At least 10 g of faeces was collected and removed from the rectum of each sampled animal. The glove was taken off the hand of the person and turned inside out, enclosing the faecal material; a piece of paper with the identification details of each animal was also put inside the glove. Then, each glove was softly pressed to remove any excess air and then closed airtight. Additionally, a sticker with the identification details of each animal was added on each glove, which was finally placed into a portable refrigerator.

The body condition score of the ewes and does was also assessed. For the uniformity of the measurements and the adherence to the published standards [10], the scoring (0–5, including half scores) was performed by a certified European veterinary specialist in small ruminant health management.

The faecal samples that were collected were stored at 8.0 to 10.0 °C using portable refrigerators.

### 2.3. Laboratory Examinations

Parasitological examinations started within 48 h after the collection of samples. Initially, 5 g of each of the individual animal faecal samples from a farm was taken and mixed to form the pooled faecal sample of the farm, which was then processed in a homogenizing blender. The usefulness of pooling ovine faecal samples as a rapid procedure for the identification of gastrointestinal parasites at farm level was confirmed by Rinaldi et al. [14]. This was followed by performing the flotation method (with zinc sulphate) in a sample (1 g) obtained from the pooled faecal sample. For this examination, the original method of Faust et al. [15] was followed, modified slightly as described by Sioutas et al. [16]. A faecal smear was performed and stained according to the Ziehl–Neelsen technique for microscopic observation [17]. Each technique was performed four times, on four different samples taken from the pooled sample. In a proportion of the farms sampled (Tables S1 and S2), the same examinations were also performed on the faecal samples from all individual animals sampled on those farms.

With regard to the modified flotation method followed [16], the details are as below. Initially, 3 g of faecal material was weighed and placed into separate 15 mL glass conical-bottom centrifuge tubes. These were filled with water, and the faecal material was homogenised. Following that, the material was filtered through a wire mesh (aperture: 250 μm) to eliminate coarse faecal debris, and the suspension was transferred into new tubes for centrifugation at 200 *g* (revolutions per minute) for 3 min. Subsequently, the supernatant was discarded, and the tubes were filled to their half with a 33% zinc sulphate solution (specific gravity: 1.34). The faecal residues were resuspended by using a wooden stick, and the tubes were then filled fully with zinc sulphate until a meniscus was formed at the top. A coverslip (18 mm × 18 mm) was placed on top of the tubes, and these were centrifuged again (130 *g* for 3 min.). Finally, the coverslip was removed and was transferred to new microscope slides for examination with a light optical microscope at 100× and 400× magnification.

The detection of a specific protozoan parasite at least once in the four times that each technique was performed, was considered to indicate the presence of the respective organism on the farm, and the farm was declared as “positive” for that particular protozoan parasite.

### 2.4. Data Management and Statistical Analysis

Data were entered into Microsoft Excel and analysed using SPSS v. 21 (IBM Analytics, Armonk, NY, USA). A basic descriptive analysis was performed, and exact binomial confidence intervals (CIs) were obtained.

In total, 31 variables (related to the infrastructure, animals, and management on the farms) were evaluated for potential association with the recovery of protozoan parasites from the pooled faecal samples (Table S3); these were either taken directly from the answers of the interview performed at the start of the visit or were calculated based on these answers. For each of these variables, categories were created according to the answers of the farmers.

In order to evaluate potential associations with the location of the farms, the 13 administrative regions of the country were clustered into four main areas: central, islands, north, and south, as detailed in Table S4 and presented in Figure S1.

The outcomes of “presence of *Eimeria* in faecal samples”, “presence of *Giardia* in faecal samples”, and “presence of *Cryptosporidium* in faecal samples” were considered. Exact binomial CIs were obtained. Separate analyses were performed for sheep flocks (*n* = 325) and goat herds (*n* = 119). Initially, standard univariable analysis was performed (Pearson’s chi-squared test and simple logistic regression). Then, multivariable models were created (mixed-effects logistic regression with flocks/herds as the random effect), with all variables that achieved a significance of *p* ≤ 0.2 in the univariable analysis (Table S5). Backwards elimination was then applied, and variables were progressively removed. The *p* value of removal of a variable was assessed by the likelihood ratio test, and for those with a *p* value of > 0.2, the variable with the largest probability was removed. This process was repeated until no variable could be removed with a *p* value of >0.2. The variables required for the final multivariable tests for each of the three outcomes are presented in Table S5.

The potential association of the presence of protozoan parasites in the faecal samples with the mean body condition score of the animals evaluated in each flock/herd was assessed by using analysis of variance. Additionally, the potential association of the presence of protozoan parasites with the average annual milk production per ewe/doe on each farm, as deduced from the answers of the farmer during the interview [8], was assessed by analysis of variance. Comparisons were made for farms on which no protozoan parasites were recovered versus farms on which at least one or all three protozoan parasites were recovered in faecal samples.

Farmers were assigned to one of two cohorts, in accordance to whether their response to the question to describe the “The two health problems in lambs/kids considered to be of the higher importance” [8], included or did not include “diarrhoea” as one of these two health problems. The association of that response with the presence of each of the three protozoan parasites in faecal samples from the respective farms was assessed with Pearson’s chi-squared test.

Statistical significance was set at *p* ≤ 0.05.

## 3. Results

### 3.1. Descriptive Results

*Eimeria* oocysts were found in faecal samples from 305 farms (68.7%; 95% CI: 64.2–72.8%), *Giardia* cysts in samples from 146 farms (32.9%; 95% CI: 28.7–37.4%), and *Cryptosporidium* oocysts in samples from 36 farms (8.1%; 95% CI: 5.9–11.0%) (Table 1). Overall, protozoan parasites were found in faecal samples from 341 farms (76.8%); all three protozoa concurrently were found in samples from 10 farms (2.3%). With regard to the results of the examination of faecal samples from individual animals, among farms on which the protozoan parasites were detected in pooled samples, the overall prevalence of detection of *Eimeria* oocysts (*n* = 90 farms) was 97.7% (95% CI: 97.0–98.2%), of *Giardia* cysts (*n* = 43 farms) was 93.9% (95% CI: 92.4–95.1%), and of *Cryptosporidium* oocysts (*n* = 16 farms) was 96.2% (95% CI: 94.2–97.5%) among the sampled animals (Tables S1 and S2).

Detailed results according to the part of the country where the farms were located are in Table 2. Differences between farms in the four area parts of the country were seen only for the presence of *Eimeria* oocysts in samples from goat herds; for those, a significantly lower prevalence was found on the islands (25.0% versus 66.0% overall in the continental part of the country).

### 3.2. Association of Recovery of Protozoan Parasites with Body Condition Score and Milk Production

No significant difference was seen in the mean body condition score of the animals on the farms on which protozoan parasites were or were not detected in faecal samples (*p* > 0.85 for sheep flocks; *p* > 0.15 for goat herds). No significant difference was seen in the average annual milk production per animal between respective farms (*p* > 0.11 for sheep flocks; *p* > 0.08 for goat herds). Detailed results are in Table 3.

### 3.3. Variables Associated with Presence of Protozoan Parasites in Faecal Samples

#### 3.3.1. Presence of *Eimeria* in Faecal Samples

The results of the univariable analysis for the presence of *Eimeria* in faecal samples from sheep are detailed in Table S6. During the multivariable analysis, only the lack of a designated building for lambs emerged as a significant factor (*p* = 0.008) (Table 4 and Table S5).

A significant association with the presence of *Eimeria* in faecal samples from goats was evident in the univariable analysis for the following five variables: the availability of a designated building for kids (*p* = 0.035), if grazing was practiced (*p* = 0.024), collaboration with a veterinarian (*p* = 0.010), the preventive use of laboratory diagnostic examinations in faecal samples (*p* = 0.036), and age of kid removal from their dams (*p* = 0.026) (Table S7). However, no variable emerged as a significant factor (*p* > 0.07) in the multivariable analysis (Table S5).

#### 3.3.2. Presence of *Giardia* in Faecal Samples

With regard to the presence of *Giardia* in faecal samples from sheep, significant associations were found neither in the univariable analysis (Table S8), nor in the multivariable analysis (*p* > 0.06) (Table S5).

The results of the univariable analysis for the presence of *Giardia* in faecal samples from goats are detailed in Table S9. During the multivariable analysis, only the lack of a main building for animals emerged as a significant factor (*p* = 0.035) (Table 5 and Table S5).

#### 3.3.3. Presence of *Cryptosporidium* in Faecal Samples

The results of the univariable analysis for the presence of *Cryptosporidium* in faecal samples from sheep are detailed in Table S10. During the multivariable analysis, the following emerged as significant factors: (a) lack of grazing of animals (*p* = 0.0002) and (b) the routine overdosing of pharmaceuticals (*p* = 0.029) (Table 6, Table S5).

The results of the univariable analysis for the presence of *Cryptosporidium* in faecal samples from goats are detailed in Table S11. During the multivariable analysis, the following emerged as significant factors: (a) the management applied on farms (*p* < 0.0001), (b) the number of female animals on the farm (*p* = 0.017), and (c) the extent of total land available to animals for grazing (*p* = 0.040) (Table 6 and Table S5).

### 3.4. Presence of Protozoan Parasites in Faecal Samples from Adult Animals and Farmers’ Perception Regarding the Significance of Diarrhoea as a Problem in Lambs or Kids

Of the 444 farmers, 316 (71.2%) declared that they considered diarrhoea as one of the two most important health problems in their lambs/kids. All three protozoan genera were found significantly more frequently in the faecal samples collected from the flocks/herds of these 316 farmers than in the samples from the farms of those who did not declare such an importance (*p* < 0.045 for all comparisons) (Table 7).

## 4. Discussion

### 4.1. Preamble

Usually, studies investigating protozoan infections in small ruminants have focused on infections in lambs and kids, animals in which *Eimeria*, *Giardia*, and *Cryptosporidium* can cause significant clinical disease, occasionally also leading to death [6,7]. In the present work, the clinical importance of these protozoan parasites was confirmed in an indirect way by the farmers, given the significant association between the recovery of protozoa from faecal samples and the declaration, by respective farmers, of lamb/kid diarrhoea as an important disease in young animals on their farms (Table 7). Despite the limitations (e.g., various other microorganisms participate as aetiological agents of diarrhoeic syndrome in lambs), there is some merit in the finding, which indicates, in an indirect way, the severity and importance of clinical disease caused by these protozoa in lambs and kids.

One may argue that the examination of pooled faecal samples rather than samples from individual animals might possibly have led to a reduced estimation of the true number of farms with the presence of protozoan parasites. Whilst there is merit to this hypothesis, for an extensive countrywide study, the issue, to some extent, was overcome by the large number of farms included in the study, as well as by performing the examination for the presence of protozoan parasites four times in the pooled faecal samples from each farm, and declaring the farm as “positive” if protozoa were detected in at least one of them. This increased the sensitivity of the method. Moreover, Rinaldi et al. [14] first confirmed the usefulness and reliability of using pooled faecal samples to detect the level of gastrointestinal parasitic infections in farms.

The present research took a different approach, which differed from most relevant studies by investigating the presence of protozoan parasites in adult animals on the farms. The study included small ruminant farms from all parts of Greece. That way, conditions prevailing throughout the country were taken into account, and factors of regional importance weighed less. In addition, in order to minimize possible bias, the study used consistent methodologies and ensured that specific tasks were always performed by the same investigators.

When no young animals are present on small ruminant farms, e.g., after slaughter, ewes and does act as carriers, who harbour the protozoan parasites and maintain the causal agents on the farm. These parasites are of little pathogenicity for adult animals, not causing clinical disease [18]. This was also confirmed clearly in the current work, as the presence of the protozoa was not associated with adverse effects in the body condition score or milk production on the respective farms (Table 3). Hence, adult animals are often forgotten as potential sources of the infective agents.

After lambing/kidding, adult animals can shed the protozoa due to the peri-parturient relaxation of immunity [19,20,21], which occurs for three weeks before and after parturition [22]. This contributes to the dissemination of high numbers of the protozoa in the farm environment and the subsequent infection of lambs/kids [18]. The increased metabolic requirements of dairy small ruminants for milk production may potentially cause some immunocompromise, thus contributing to easier infection of lactating adult females.

Of the available techniques for the detection of these protozoa, the microscopic detection of the parasitic forms in faecal samples is a reliable means to confirm the presence of active infections on the farms in the study. However, it is laborious and time-consuming, but on the other hand, for such an extensive investigation, where cost was an important determinant, it provided a well-priced means. Certainly, antigen detection molecular-based tests are more sensitive and less laborious than microscopic observation. However, their findings do not provide results exclusively for active infections, and they are far more costly than microscopic observation, especially in the present study, in which a large number of samples was processed.

### 4.2. Predictors for Occurrence of Protozoan Parasites in Adult Ewes and Does

The analysis of management variables provided novel evidence regarding factors that could be significant for the occurrence of protozoan infections in adult small ruminants. Limiting protozoan presence in adult animals would contribute to the reduction of cases of disease in young ones. Given the threat for the emergence of resistance to antiprotozoal drugs [23,24,25], this would contribute, at least to some extent, to the control of relevant infections in lambs and kids.

A significant difference was noted between sheep and goats in the detection of *Eimeria* in the faecal samples, with a smaller recovery rate seen in goat herds (Table 1). The relevant international evidence is conflicting; for example, Etsay et al. [26], who worked in Ethiopia, reported results similar to ours, i.e., a higher recovery rate of *Eimeria* for faecal samples of sheep than for those of goats, whilst Mahamaden et al. [27] reported totally different findings from work performed in Egypt.

These variations can be the result of differences in sampling protocols, in climatic conditions, and in the breeds of animals included in the present studies, among others. All those would be reflected in the infectivity of coccidian oocysts or the susceptibility of animals sampled. Another hypothesis suggests that this may possibly be the result of differences in the grazing mode and in the ingestive behaviour that occur between the two animal species, in particular, differences in selectivity of feed. Sheep forage mainly grasses rather than shrubs (60% and 10%, respectively, of the total forage intake), whilst, in contrast, goats prefer to forage primarily shrubs instead of grasses (60% and 20%, respectively, of the total forage intake) [28,29]. This is the result of the anatomical adaptations of the mouths of sheep and goats, which allow the respective type of grazing [30]. All the above might have contributed to sheep ingesting *Eimeria* oocysts (which are found at soil level rather than on leaves) more easily than goats, resulting in more frequent infections in the former animals.

There was also a significantly lower recovery rate of *Eimeria* from farms on the islands of the country (Table 2). This could reflect the significantly lower precipitation that occurred in the locations of small ruminant farms on the islands (on average, 1.52 mm annually versus 1.58 mm in locations in other parts of the country [31]). As the result of lower precipitation, the soil in those locations was less wet and humid, leading to reduced sporulation and infectivity of the oocysts. In this hypothesis, the reduced infectivity of oocysts was reflected in the reduced recovery of *Eimeria* oocysts from samples collected from farms on the islands.

The presence of a designated building for lambs as a predictor for the identification of *Eimeria* in faecal samples from ewes (Table 4) can be associated with a reduced exposure of animals to infection, as adults and young animals would not share the same animal houses continuously. Oocysts excreted by adult animals would be the initial source of infection on a farm; subsequently, due to the high multiplication rate of the protozoa, infected lambs/kids would excrete particularly high numbers of oocysts into the environment [32], and that way, the excretion of oocysts from lambs/kids rapidly becomes the main source of infection for other animals on the farm [18]. Then, the infection can spread rapidly within a farm of susceptible animals [33,34,35]. Infected lambs housed indoors can contaminate the animal house environment with oocysts. If lambs are separated at an early age from their dams, this cycle would break, resulting in lower infection rates in the adult females.

No significant predictor could be identified for the presence of *Giardia* in faecal samples from sheep. Moreover, the identification of the lack of a main building on the farm as the only significant predictor for the presence of *Giardia* cysts in samples from goats (Table 5) could not be associated with a plausible explanation of clinical significance. In any case, the difficulties associated with an accurate *Giardia* diagnosis, arising from the intermittent excretion of the parasite [36,37], present challenges for the accurate detection of infection status.

For the presence of *Cryptosporidium*, it should first be mentioned that the prevalence of the infection was found to be at the lower end of the relevant studies internationally, and certainly lower than the mean cross-sectional prevalence (~30% for sheep and ~15% for goats) as presented by Robertson [3]. A probable reason for this significant discrepancy could be the different farming systems prevailing in Greece, where only a few farms are managed under the intensive system. The management system (intensive or semi-intensive) was identified as the most important predictor for the presence of *Cryptosporidium* in faecal samples from goats, whilst a lack of grazing (which is the main feature of the intensive management system) was identified as the most important predictor for the presence of *Cryptosporidium* in faecal samples from sheep (Table 6). These findings are in line with the mode of transmission of the organism, which is facilitated in high-density animal houses [38]. With regard to the other variables identified as predictors for infection, the overdosing of pharmaceuticals (including antiprotozoal preparations) potentially might have contributed to the development of resistance by strains of the organism. This, in turn, has led to the spread of the pathogen to the adult animals within a farm, which can act as carriers for transmitting the organism to newborn lambs.

As a whole, the results show a tendency for practices related to intensive management in farms (e.g., reduced grazing and a high number of animals) to be associated with the recovery of the protozoa from faecal samples. This is reasonable, given the mode of dissemination of the pathogens, which is facilitated in conditions of overstocking and close contact between animals.

### 4.3. Zoonotic Significance of Findings

In relation to the zoonotic significance of the findings, one should always have in mind that whilst *Eimeria* protozoa have host species specificity, i.e., sheep and goats are of no importance to public health, *Giardia* and *Cryptosporidium* can infect humans. The infection of people occurs by various means, e.g., close contact with infected individuals, consumption of infected food or water, or contact with infected animals or environments contaminated with animal faeces. Both parasites are included in the standard differential diagnosis of intestinal infections in humans, and the various rapid detection methods (e.g., nucleic acid amplification methods) include these two protozoa in the array of pathogens for in vitro detection and identification (e.g., the “BioFire^®^ FilmArray^®^ Gastrointestinal (GI) Panel”) [39]. In contrast, sheep and goats do not pose a real risk for the infection of people with *Eimeria*, as these protozoa have host species specificity; however, infections by these protozoa have a tremendous financial importance in agriculture worldwide.

Thus, it is important to know the infection status of the adult animals in a flock or herd with these protozoa, as throughout the annual production cycle of small ruminants, there are many opportunities for the infection of people working on the farm, for example, in the milking parlour, where milkers come in close contact with the faecal excreta of animals during the milking routine.

## 5. Conclusions

The study explored the importance of protozoan parasites in adult small ruminants, an underexamined topic internationally. The findings indicated the presence of the protozoa in adult sheep and goats. These animals can be important sources of infection for newborn animals immediately after lambing/kidding, when, due to the peri-parturient relaxation of immunity, the faecal excretion of parasitic forms increases, in that way increasing the risk for infecting newborns. Although these infections remain asymptomatic for adult animals, the dissemination of the pathogens in the environment of the farms poses a significant threat to newborns, in which all three protozoa can cause severe, and potentially lethal, disease.

The present findings should be taken into account on farms with problems of protozoan infections in lambs or kids. The management factors that emerged to be of significance can be considered, in order to control the infection, by reducing the presence of the infections in the adult animals, which are the source of infection for the young ones. Moreover, on farms where these factors apply as standard practices (e.g., intensive management system), it is worth it to apply specific preventive measures against the respective infections.

## Figures and Tables

**Table 1:** Presence of protozoan parasites in pooled faecal samples from 444 small ruminant farms in a countrywide investigation (April 2019 to July 2020) in Greece.

	Number of Farms with Samples in Which Protozoan Parasites Were Detected (Proportion of These Farms among All Farms in the Study)			
Protozoan Parasite	All Farms	Sheep Flocks	Goat Herds	*p* Value ^1^
At least one parasite	341 (76.8%)	256 (78.8%)	85 (71.4%)	0.10
*Eimeria*	305 (68.7%)	233 (71.7%)	72 (60.5%)	0.024
*Giardia*	146 (32.9%)	106 (32.6%)	40 (33.6%)	0.84
*Cryptosporidium*	36 (8.1%)	23 (7.1%)	13 (10.9%)	0.19
All three parasites	10 (2.3%)	6 (1.8%)	4 (3.4%)	0.34

^1^ *p* value for comparison between frequencies in sheep farms and goat farms.

**Table 2:** Presence of protozoan parasites in pooled faecal samples from 444 small ruminant farms in a countrywide investigation (April 2019 to July 2020) in Greece, in accordance with the part of the country where the farms were located (number of farms in samples in which protozoan parasites were detected (proportion of these farms among all farms in the study)).

Location of Farms (Part of Country)	*n*		*Eimeria*		*Giardia*		*Cryptosporidium*	
	S ^1^	G ^1^	Sheep Farms	Goat Farms	Sheep Farms	Goat Farms	Sheep Farms	Goat Farms
Central part	127	36	90 (70.9%)	20 (55.6%)	40 (31.5%)	12 (33.3%)	11 (8.7%)	6 (16.7%)
Islands	42	16	28 (66.7%)	4 (25.0%)	13 (31.0%)	6 (37.5%)	1 (2.4%)	1 (6.3%)
Northern part	88	36	68 (77.3%)	28 (77.8%)	27 (30.7%)	13 (36.1%)	8 (9.1%)	5 (13.9%)
Southern part	68	31	47 (69.1%)	20 (64.5%)	26 (38.2%)	9 (29.0%)	3 (4.4%)	1 (3.2%)
*p* ^2^			0.54	0.004	0.74	0.92	0.37	0.28

^1^ S = sheep farms/G = goat farms; ^2^ *p* for comparison between frequencies in farms in the various parts of the country.

**Table 3:** Association of presence of protozoan parasites in pooled faecal samples with body condition score and milk production (mean ± standard error of the mean) on 444 small ruminant farms in a countrywide investigation (April 2019 to July 2020) in Greece.

Recovery of Protozoan Parasites	Sheep Flocks		Goat Herds	
	Body Condition Score	Average Milk Production per Ewe (mL)	Body Condition Score	Average Milk Production per Doe (mL)
No recovery of any parasite	2.38 ± 0.05	193 ± 10	2.49 ± 0.06	187 ± 18
Recovery of at least one parasite	2.38 ± 0.02	211 ± 6	2.57 ± 0.03	206 ± 13
Recovery of all three parasites	2.35 ± 0.12	253 ± 44	2.66 ± 0.03	290 ± 72

**Table 4:** Results of multivariable analysis for the presence of *Eimeria* in faecal samples from 325 sheep flocks in Greece.

Variable	Odds Ratio ^1^ (95% Confidence Intervals)	*p* Value
Availability of a designated building for lambs		0.008
Yes (*n* = 243)	Reference	-
No (*n* = 82)	2.296 (1.217–4.334)	0.010

^1^ odds ratio calculated against the lowest prevalence associations of the variable.

**Table 5:** Results of multivariable analysis for the presence of *Giardia* in faecal samples from 119 goat herds.

Variables	Odds Ratio ^1^ (95% Confidence Intervals)	*p* Value
Availability of a main building for animals		0.035
Yes (*n* = 117)	Reference	-
No (*n* = 2)	10.325 (0.484–220.356)	0.13

^1^ odds ratio calculated against the lowest prevalence associations of the variable.

**Table 6:** Results of multivariable analysis for the presence of *Cryptosporidium* in faecal samples from ewes and does in 325 sheep flocks and 119 goat herds, respectively.

Variables	Odds Ratio ^1^ (95% Confidence Intervals)	*p* Value
Sheep flocks		
Grazing practiced		0.0002
Yes (*n* = 281)	Reference	-
No (*n* = 44)	11.365 (4.601–28.073)	<0.0001
Routine overdosing of pharmaceuticals		0.029
Yes (*n* = 61)	2.009 (0.788–5.122)	0.14
No (*n* = 264)	-	-
Goat herds		
Management system applied on farm		<0.0001
Intensive	228.429 (10.588–4928.308)	0.0005
Semi-intensive	41.000 (2.249–747.534)	0.012
Semi-extensive	Reference	-
Extensive	3.000 (0.058–156.078)	0.59
No. of female animals on the farm		0.017
0–165 animals	Reference	-
166–330 animals	1.205 (0.253–5.725)	0.82
331–500 animals	8.281 (1.829–37.503)	0.006
>500 animals	1.104 (0.113–10.786)	0.93
Total land available for grazing (acres per animal)		0.04
0–0.5	11.200 (3.145–39.887)	0.0002
>0.5	Reference	

^1^ odds ratio calculated against the lowest prevalence associations of the variable.

**Table 7:** Proportion of farms with recovery of protozoan parasites from the faecal samples of animals, as found during a countrywide investigation (April 2019 to July 2020) in Greece, in accordance with the farmers’ perception of the significance of diarrhoea as a problem in lambs or kids on their farms.

Protozoan Parasite	Farmers Who Considered Diarrhoea as a Significant Problem in Lambs/Kids	Farmers Who Did Not Consider Diarrhoea as a Significant Problem in Lambs/Kids	*p* Value ^1^
*n*	316	128	
*Eimeria*	229/316 (72.5%)	76/128 (59.4%)	0.007
*Giardia*	113/316 (35.8%)	33/128 (25.8%)	0.043
*Cryptosporidium*	32/316 (10.1%)	4/128 (3.1%)	0.014

^1^ *p* value for comparison of frequencies between two categories of farmers.

## Data Availability

Most data presented in this study are in the Supplementary Materials. The remaining data are available upon request from the corresponding author. The data are not publicly available, as they form part of the PhD thesis of the first author, which has not yet been examined, approved, and uploaded in the official depository of Ph.D. theses from Greek universities.

## Supplementary Materials

The following are available online at https://www.mdpi.com/article/10.3390/microorganisms10101931/s1, Table S1: Presence of protozoan parasites in faecal samples from individual animals on small ruminant farms, in which protozoa had been found in pooled faecal samples, in Greece. Table S2: Presence of protozoan parasites in faecal samples from individual animals on small ruminant farms, in which protozoa had not been found in pooled faecal samples, in Greece. Table S3: Variables (*n* = 31) evaluated for potential association with presence of protozoan parasites in faecal samples from 444 small ruminant farms in Greece. Table S4: Geographical areas of Greece (*n* = 4), in which administrative regions and regional units of the country were clustered, for characterizing location of 444 small ruminant farms from which faecal samples were collected. Table S5: Details of multivariable models employed for the evaluation of the presence of protozoan parasites in faecal samples from 444 small ruminant farms in Greece. Table S6: Associations of presence of *Eimeria* in faecal samples from 325 sheep flocks in Greece, as found in univariable analysis. Table S7: Associations of presence of *Eimeria* in faecal samples from 119 goat herds in Greece, as found in univariable analysis. Table S8: Associations of presence of *Giardia* in faecal samples from 325 sheep flocks in Greece, as found in univariable analysis. Table S9: Associations of presence of *Giardia* in faecal samples from 119 goat herds in Greece, as found in univariable analysis. Table S10: Associations of presence of *Cryptosporidium* in faecal samples from 325 sheep flocks in Greece, as found in univariable analysis. Table S11: Associations of presence of *Cryptosporidium* in faecal samples from 119 goat herds in Greece, as found in univariable analysis. Figure S1: Map of Greece indicating the geographic areas of the country (*n* = 4), in which administrative regions and regional units of the country were clustered, for characterizing location of 444 small ruminant farms from which faecal samples were collected.
