# Supplementary material for: Protozoan Parasites in Adult Dairy Small Ruminants and Potential Predictors for Their Presence in Faecal Samples
Source: Microorganisms. 2022 Sep 28;10(10):1931. doi: 10.3390/microorganisms10101931 (PMC9611716; doi:10.3390/microorganisms10101931)
Supplement: Supplementary file 1 [file microorganisms-10-01931-s001.zip › microorganisms-1887924-supplementary.pdf]

Protozoan Parasites in Dairy Small Ruminants and Potential Risk Factors for their Presence in Faecal Samples

Daphne T. Lianou, Konstantinos Arsenopoulos, Charalambia K. Michael, Elias Papadopoulos, and George C. Fthenakis

Table S1. Presence of protozoan parasites in faecal samples from individual animals in small ruminant farms, in which protozoa had been found in pooled faecal samples, in Greece.

Protozoan parasites	No. of farms in which protozoa were found in pooled faecal samples and faecal samples from individual animals were also examined	No. of animals present in these farms	No. of animals sampled in these farms	No. of animals in the faecal samples of which protozoa were found	Overall prevalence of recovery of protozoa in samples from individual animals	Among farms median prevalence of recovery of protozoa in samples from individual animals
			Sheep flocks			
Eimeria	65 (27.9% [1])	16,041	1,830	1,807	98.7%	100.0%
Giardia	35 (33.0%)	8,256	980	916	93.5%	93.3%
Cryptosporidium	11 (47.8%)	4,758	360	346	96.1%	96.7%
			Goats herds			
Eimeria	25 (34.7%)	6,497	760	723	95.1%	95.0%
Giardia	8 (20.0%)	1,769	230	220	95.7%	95.0%
Cryptosporidium	5 (38.5%)	2,300	190	183	96.3%	95.0%

[1] figures in brackets indicate the proportion of these farms among all those from which protozoa had been found in pooled faecal samples.

Table S2. Presence of protozoan parasites in faecal samples from individual animals in small ruminant farms, in which protozoa had not been found in pooled faecal samples, in Greece.

Protozoan parasites	No. of farms in which protozoa were not found in pooled faecal samples and faecal samples from individual animals were also examined	No. of animals present in these farms	No. of animals sampled in these farms	No. of animals in the faecal samples of which protozoa were found	Overall prevalence of recovery of protozoa in samples from individual animals	Among farms median prevalence of recovery of protozoa in samples from individual animals
			Sheep flocks			
Eimeria	22 (23.9% [1])	5,636	630	0	0.0%	0.0%
Giardia	55 (25.1%)	14,261	1,570	0	0.0%	0.0%
Cryptosporidium	80 (26.5%)	19,409	2,240	0	0.0%	0.0%
			Goats herds			
Eimeria	8 (17.0%)	1,255	200	0	0.0%	0.0%
Giardia	25 (31.6%)	5,983	730	0	0.0%	0.0%
Cryptosporidium	31 (29.2%)	6,842	880	0	0.0%	0.0%

[1] figures in brackets indicate the proportion of these farms among all those from which protozoa had not been found in pooled faecal samples.

Table S3. Variables (n = 31) evaluated for potential association with presence of protozoan parasites in faecal samples from 444 small ruminant farms in Greece.

Management system applied in the farm (description according to EFSA classification)
Years at the location (no. of years)
Month into the lactation period at sampling (month)
Availability of a main building for animals (yes / no)
Availability of a designated building for lambs / kids (yes / no)
Availability of a designated lambing / kidding area (yes / no)
Availability of a lavatory (yes / no)
Material of the floor of the barn (soil / other)
Availability of straw bedding (yes / no)
Annual frequency of removal / clean-up of the straw bedding (no. of occasions)
Grazing practiced (yes / no)
Grazing land available to animals (acres per animal)
No. of female animals in the farm (no.)
Breed of ewes /does (description)
Month of the start of the lambing / kidding season (description)
Collaboration with a veterinarian (yes / no)
Use of laboratory diagnostic examinations in faecal samples preventively (yes / no)
Means of calculating live bodyweight for the administration of pharmaceutical products (weighing / estimation)
Routine overdosing (compared to dose prescribed) of pharmaceuticals (yes / no)
Total visits made annually by veterinarians to the farm during the preceding season (no.)
Application of reproductive control practices in the farm (yes / no)
Newborn care and specific monitoring (yes / no)
Lamb / kid fostering to female animals other than their dams (yes / no)
Administration of a lamb- / kid-specific diet (yes / no)
Age of lamb / kid removal from their dams (days)
Seasonal transfer of animals to other site (yes / no)
Manure management (spread in the fields / removed otherwise)
Annual frequency of systemic disinfections in the farm (no.)
Shearing of animals (yes / no)
Provision of finished feed (concentrate) to animals throughout the year (yes / no)
Provision of finished feed (concentrate) to young animals (yes / no)

Table S4. Geographical areas of Greece ($n = 4$), in which administrative regions and regional units of the country were clustered, for characterizing location of 444 small ruminant farms from which faecal samples were collected.

**Central**
Region of Central Greece
Region of Thessaly
From the region of Epirus: regional unit of Arta and regional unit of Preveza
From the region of Western Greece: regional unit of Aetolia-Acarnania
**Islands**
Region of Crete
Region of Ionian islands
Region of North Aegean
Region of South Aegean
**North**
Region of Central Macedonia
Region of Eastern Maecedonia and Thrace
Region of Western Macedonia
From the region of Epirus: regional unit of Ioannina and regional unit of Thesprotia
**South**
Region of Attica
Region of Peloponnese
Region of Western Macedonia
From the region of Western Greece: regional unit of Achaea and regional unit of Elis

Figure S1. Map of Greece indicating the geographic areas of the country (*n* = 4), in which administrative regions and regional units of the country were clustered, for characterizing location of 444 small ruminant farms from which faecal samples were collected (blue: Central, green: Islands, yellow: North, red: South).

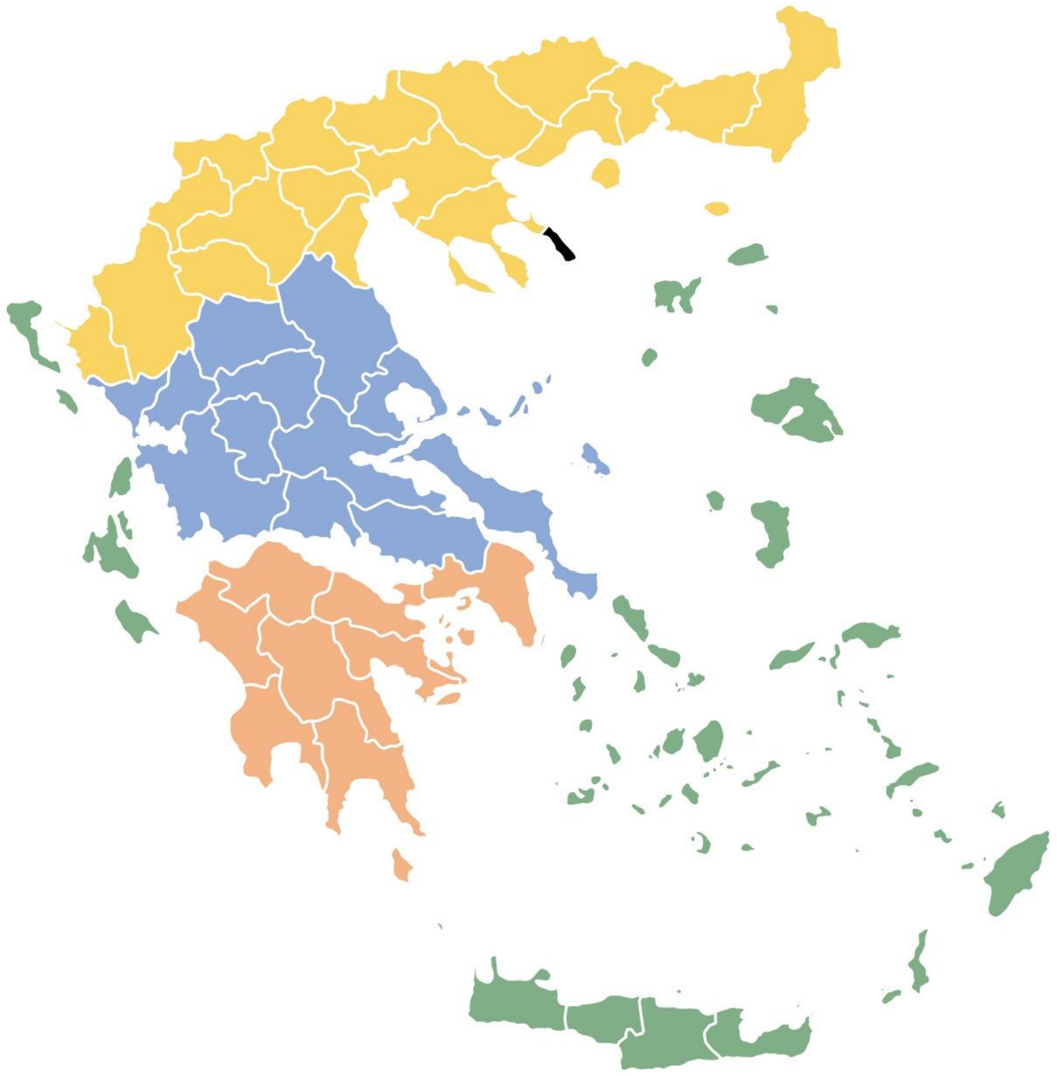

Table S5. Details of multivariable models employed for the evaluation of the presence of protozoan parasites in faecal samples from 444 small ruminant farms in Greece.

Outcome	Variables offered to the multivariable models (*n*)	Variables required in the final models
Presence of *Eimeria* in faecal samples – sheep flocks	5	(a) Availability of a designated building for lambs, (b) Collaboration with a veterinarian, (c) Means of calculating live bodyweight for the administration of pharmaceutical products
Presence of *Eimeria* in faecal samples – goat herds	9	(a) Availability of a designated building for kids, (b) Grazing practiced, (c) Collaboration with a veterinarian, (d) Means of calculating live bodyweight for the administration of pharmaceutical products, (e) Age of kid removal from their dams
Presence of *Giardia* in pooled faecal samples – sheep flocks	5	(a) Years at the location, (b) Grazing practiced, (c) Application of reproductive control practices in the farm
Presence of *Giardia* in pooled faecal samples – goat herds	3	(a) Availability of a main building for animals, (b) Application of reproductive control practices in the farm
Presence of *Cryptosporidium* in faecal samples – sheep flocks	13	(a) Management system applied in the farm, (b) Years at the present location, (c) Availability of a designated building for lambs, (d) Grazing practiced, (e) Breed of ewes, (f) Routine overdosing of pharmaceuticals, (g) Lamb fostering to female animals other than their dams, (h) Annual frequency of systemic disinfections in the farm
Presence of *Cryptosporidium* in faecal samples – goat herds	13	(a) Management system applied in the farm, (b) Availability of straw bedding, (c) Annual frequency of removal / clean-up of the straw bedding, (d) Grazing practiced, (e) Means of calculating live bodyweight for the administration of pharmaceutical products, (f) Application of reproductive control practices in the farm, (g) Kid fostering to female animals other than their dams

Table S6. Associations of presence of *Eimeria* in faecal samples from 325 sheep flocks in Greece, as found in univariable analysis.

<table>
<tr><th colspan="24">Number of farms</th><th rowspan="2">p</th></tr>
<tr><th colspan="12">Farms with presence of Eimeria in faecal samples (n = 233)</th><th colspan="12">Farms with no presence of Eimeria in faecal samples (n = 92)</th></tr>
<tr><td colspan="24">Management system applied in the farm</td><td></td></tr>
<tr><td colspan="3">Intensive</td><td colspan="3">Semi-intensive</td><td colspan="3">Semi-extensive</td><td colspan="3">Extensive</td><td colspan="3">Intensive</td><td colspan="3">Semi-intensive</td><td colspan="3">Semi-extensive</td><td colspan="3">Extensive</td><td></td></tr>
<tr><td colspan="3">35/43</td><td colspan="3">104/151</td><td colspan="3">76/107</td><td colspan="3">18/24</td><td colspan="3">8/43</td><td colspan="3">47/151</td><td colspan="3">31/107</td><td colspan="3">6/24</td><td>0.43</td></tr>
<tr><td colspan="24">Years at the present location</td><td></td></tr>
<tr><td colspan="4">0 – 2</td><td colspan="4">3 – 5</td><td colspan="4">> 5</td><td colspan="4">0 – 2</td><td colspan="4">3 – 5</td><td colspan="4">> 5</td><td></td></tr>
<tr><td colspan="4">4/5</td><td colspan="4">29/40</td><td colspan="4">200/280</td><td colspan="4">1/5</td><td colspan="4">11/40</td><td colspan="4">80/280</td><td>0.91</td></tr>
<tr><td colspan="24">Month into the lactation period at sampling</td><td></td></tr>
<tr><td colspan="3">0–1st</td><td colspan="3">2nd–5th</td><td colspan="3">6th–9th</td><td colspan="3">After 9th</td><td colspan="3">0–1st</td><td colspan="3">2nd–5th</td><td colspan="3">6th–9th</td><td colspan="3">After 9th</td><td></td></tr>
<tr><td colspan="3">16/23</td><td colspan="3">93/138</td><td colspan="3">109/147</td><td colspan="3">15/17</td><td colspan="3">7/23</td><td colspan="3">45/138</td><td colspan="3">38/147</td><td colspan="3">2/17</td><td>0.26</td></tr>
<tr><td colspan="24">Availability of a main building for animals</td><td></td></tr>
<tr><td colspan="6">Yes</td><td colspan="6">No</td><td colspan="6">Yes</td><td colspan="6">No</td><td></td></tr>
<tr><td colspan="6">229/318</td><td colspan="6">4/7</td><td colspan="6">89/318</td><td colspan="6">3/7</td><td>0.39</td></tr>
<tr><td colspan="24">Availability of a designated building for lambs</td><td></td></tr>
<tr><td colspan="6">Yes</td><td colspan="6">No</td><td colspan="6">Yes</td><td colspan="6">No</td><td></td></tr>
<tr><td colspan="6">165/243</td><td colspan="6">68/82</td><td colspan="6">78/243</td><td colspan="6">14/82</td><td>0.009</td></tr>
<tr><td colspan="24">Availability of a designated lambing area</td><td></td></tr>
<tr><td colspan="6">Yes</td><td colspan="6">No</td><td colspan="6">Yes</td><td colspan="6">No</td><td></td></tr>
<tr><td colspan="6">123/173</td><td colspan="6">110/152</td><td colspan="6">50/173</td><td colspan="6">42/152</td><td>0.80</td></tr>
<tr><td colspan="24">Availability of a lavatory</td><td></td></tr>
<tr><td colspan="6">Yes</td><td colspan="6">No</td><td colspan="6">Yes</td><td colspan="6">No</td><td></td></tr>
<tr><td colspan="6">97/143</td><td colspan="6">136/182</td><td colspan="6">46/143</td><td colspan="6">46/182</td><td>0.17</td></tr>
<tr><td colspan="24">Material of the floor of the barn</td><td></td></tr>
<tr><td colspan="6">Soil</td><td colspan="6">Other</td><td colspan="6">Soil</td><td colspan="6">Other</td><td></td></tr>
<tr><td colspan="6">208/292</td><td colspan="6">25/33</td><td colspan="6">84/292</td><td colspan="6">8/33</td><td>0.58</td></tr>
<tr><td colspan="24">Availability of straw bedding</td><td></td></tr>
<tr><td colspan="6">Yes</td><td colspan="6">No</td><td colspan="6">Yes</td><td colspan="6">No</td><td></td></tr>
<tr><td colspan="6">189/268</td><td colspan="6">44/57</td><td colspan="6">79/268</td><td colspan="6">13/57</td><td>0.31</td></tr>
<tr><td colspan="24">Annual frequency of removal / clean-up of the straw bedding</td><td></td></tr>
<tr><td colspan="6">1 – 2</td><td colspan="6">> 2</td><td colspan="6">1 – 2</td><td colspan="6">> 2</td><td></td></tr>
<tr><td colspan="6">114/166</td><td colspan="6">75/102</td><td colspan="6">52/166</td><td colspan="6">27/102</td><td>0.40</td></tr>
<tr><td colspan="24">Grazing practiced</td><td></td></tr>
<tr><td colspan="6">Yes</td><td colspan="6">No</td><td colspan="6">Yes</td><td colspan="6">No</td><td></td></tr>
<tr><td colspan="6">199/281</td><td colspan="6">34/44</td><td colspan="6">82/281</td><td colspan="6">10/44</td><td>0.38</td></tr>
<tr><td colspan="24">Grazing land available to animals</td><td></td></tr>
<tr><td colspan="6">0 – 0.5 acre per animals</td><td colspan="6">> 0.5 acre per animal</td><td colspan="6">0 – 0.5 acre per animals</td><td colspan="6">> 0.5 acre per animal</td><td></td></tr>
<tr><td colspan="6">48/74</td><td colspan="6">151/207</td><td colspan="6">26/74</td><td colspan="6">56/207</td><td>0.19</td></tr>
</table>

No. of female animals in the farm								
0 – 165	166 – 330	331 – 500	> 500	0 – 165	166 – 330	331 – 500	> 500	
70/88	78/120	49/66	36/51	18/88	42/120	17/66	15/51	0.13
Breed of ewes								
Cross-breeds	Greek breeds	Imported breeds	Cross-breeds	Greek breeds	Imported breeds			
29/43	105/143	99/139	14/43	38/143	40/139			0.74
Month of the start of the lambing season								
All year	Autumn	Winter	Spring-Summer	All year	Autumn	Winter	Spring-Summer	
15/18	170/245	43/55	5/7	3/18	75/245	12/55	2/7	0.39
Collaboration with a veterinarian								
Yes	No	Yes	No					
203/277	30/48	74/277	18/48					0.13
Use of laboratory diagnostic examinations in faecal samples preventively								
Yes	No	Yes	No					
25/34	208/291	9/34	83/291					0.80
Means of calculating live bodyweight for the administration of pharmaceutical products								
Weighing	Estimation	Weighing	Estimation					
46/73	187/252	27/73	65/252					0.06
Routine overdosing (compared to dose prescribed) of pharmaceuticals								
Yes	No	Yes	No					
45/61	188/264	16/61	76/264					0.69
Total visits made annually by veterinarians to the farm during the preceding season								
0 – 4	5 – 7	> 7	0 – 4	5 – 7	> 7			
99/139	61/86	73/100	40/139	25/86	27/100			0.94
Application of reproductive control practices in the farm								
Yes	No	Yes	No					
73/101	160/224	28/101	64/224					0.88
Newborn care and specific monitoring								
Yes	No	Yes	No					
209/293	24/32	84/293	8/32					0.66
Lamb fostering to female animals other than their dams								
Yes	No	Yes	No					
157/221	76/104	64/221	28/104					0.70
Administration of a lamb-specific diet								
Yes	No	Yes	No					
161/226	72/99	65/226	27/99					0.78

Age of lamb removal from their dams						
< 45 days	45 – 60 days	> 60 days	< 45 days	45 – 60 days	> 60 days	
85/119	121/170	27/36	34/119	49/170	9/36	0.90

Seasonal transfer of animals to other site				
Yes	No	Yes	No	
33/49	200/276	16/49	76/276	0.46
Manure management				
Spread in the fields	Removed otherwise	Spread in the fields	Removed otherwise	
230/320	3/5	90/320	2/5	0.56
Annual frequency of systemic disinfections in the farm				
0 – 9	> 9	0 – 9	> 9	
209/293	24/32	84/293	8/32	0.66
Shearing of animals				
Yes	No	Yes	No	
228/319	5/6	91/319	1/6	0.52
Provision of finished feed (concentrate) to animals throughout the year				
Yes	No	Yes	No	
216/304	17/21	88/304	4/21	0.33
Provision of finished feed (concentrate) to young animals				
Yes	No	Yes	No	
185/255	48/70	70/255	22/70	0.51

Table S7. Associations of presence of *Eimeria* in faecal samples from 119 goat herds in Greece, as found in univariable analysis.

Number of farms								*p*
**Farms with presence of *Eimeria* in faecal samples (*n* = 72)**				**Farms with no presence of *Eimeria* in faecal samples (*n* = 47)**				
Management system applied in the farm								
Intensive	Semi-intensive	Semi-extensive	Extensive	Intensive	Semi-intensive	Semi-extensive	Extensive	
3/9	16/29	39/61	14/20	6/9	13/29	22/61	6/20	0.24
Years at the present location								
0 – 2	3 – 5	> 5		0 – 2	3 – 5	> 5		
1/1	9/15	62/103		0/1	6/15	41/103		0.72
Month into the lactation period at sampling								
0–1st	2nd–5th	6th–9th	After 9th	0–1st	2nd–5th	6th–9th	After 9th	
4/8	43/60	22/43	3/8	4/8	17/60	21/43	5/8	0.08
Availability of a main building for animals								
Yes	No			Yes	No			
71/117	1/2			46/117	1/2			0.76
Availability of a designated building for kids								
Yes	No			Yes	No			
47/86	25/33			39/86	8/33			0.035
Availability of a designated kidding area								
Yes	No			Yes	No			
34/58	38/61			24/58	23/61			0.68
Availability of a lavatory								
Yes	No			Yes	No			
20/38	52/81			18/38	29/81			0.23
Material of the floor of the barn								
Soil	Other			Soil	Other			
65/105	7/14			40/105	7/14			0.39
Availability of straw bedding								
Yes	No			Yes	No			
43/76	29/43			33/76	14/43			0.24
Annual frequency of removal / clean-up of the straw bedding								
1 – 2	> 2			1 – 2	> 2			
25/42	18/34			17/42	16/34			0.56
Grazing practiced								
Yes	No			Yes	No			
71/113	1/6			42/113	5/6			0.024

Grazing land available to animals								
0 – 0.5 acre per animals	> 0.5 acre per animal	0 – 0.5 acre per animals	> 0.5 acre per animal					
7/11	64/102	4/11	38/102					0.95
No. of female animals in the farm								
0 – 165	166 – 330	331 – 500	> 500	0 – 165	166 – 330	331 – 500	> 500	
32/56	22/37	8/13	10/13	24/56	15/37	5/13	3/13	0.62
Breed of does								
Cross-breeds	Greek breeds	Imported breeds	Cross-breeds	Greek breeds	Imported breeds			
8/18	36/56	28/45	10/18	20/56	17/45			0.31
Month of the start of the kidding season								
All year	Autumn	Winter	Spring-Summer	All year	Autumn	Winter	Spring-Summer	
4/8	37/63	29/44	2/4	4/8	26/63	15/44	2/4	0.76
Collaboration with a veterinarian								
Yes	No	Yes	No					
66/101	6/18	35/101	12/18					0.010
Use of laboratory diagnostic examinations in faecal samples preventively								
Yes	No	Yes	No					
8/16	64/103	8/16	39/103					0.036
Means of calculating live bodyweight for the administration of pharmaceutical products								
Weighing	Estimation	Weighing	Estimation					
13/27	59/92	14/27	33/92					0.14
Routine overdosing (compared to dose prescribed) of pharmaceuticals								
Yes	No	Yes	No					
14/26	58/93	12/26	35/93					0.43
Total visits made annually by veterinarians to the farm during the preceding season								
0 – 4	5 – 7	> 7	0 – 4	5 – 7	> 7			
25/51	18/25	29/43	26/51	7/25	14/43			0.08
Application of reproductive control practices in the farm								
Yes	No	Yes	No					
9/17	63/102	8/17	39/102					0.49
Newborn care and specific monitoring								
Yes	No	Yes	No					
66/109	6/10	43/109	4/10					0.97
Kid fostering to female animals other than their dams								
Yes	No	Yes	No					
49/86	23/33	37/86	10/33					0.21

Administration of a kid-specific diet						
Yes		No	Yes		No	
51/79		21/40	28/79		19/40	0.21
Age of kid removal from their dams						
< 45 days	45 – 60 days	> 60 days	< 45 days	45 – 60 days	> 60 days	
11/26	25/44	36/49	15/26	19/44	13/49	0.026
Seasonal transfer of animals to other site						
Yes		No	Yes		No	
16/28		56/91	12/28		35/91	0.68
Manure management						
Spread in the fields		Removed otherwise	Spread in the fields		Removed otherwise	
66/109		6/10	43/109		4/10	0.97
Annual frequency of systemic disinfections in the farm						
0 – 9		> 9	0 – 9		> 9	
62/100		10/19	38/100		9/19	0.44
Shearing of animals						
Yes		No	Yes		No	
65/102		7/17	37/102		10/17	0.08
Provision of finished feed (concentrate) to animals throughout the year						
Yes		No	Yes		No	
61/103		11/16	42/103		5/16	0.47
Provision of finished feed (concentrate) to young animals						
Yes		No	Yes		No	
48/79		24/40	31/79		16/40	0.94

Table S8. Associations of presence of *Giardia* in faecal samples from 325 sheep flocks in Greece, as found in univariable analysis.

**Number of farms**								***p***
**Farms with presence of *Giardia* in faecal samples (n = 106)**				**Farms with no presence of *Giardia* in faecal samples (n = 219)**				
Management system applied in the farm								
Intensive	Semi-intensive	Semi-extensive	Extensive	Intensive	Semi-intensive	Semi-extensive	Extensive	
8/43	59/151	32/107	7/24	35/43	92/151	75/107	17/24	0.07
Years at the present location								
0 – 2	3 – 5	> 5		0 – 2	3 – 5	> 5		
2/5	18/40	86/280		3/5	22/40	194/280		0.18
Month into the lactation period at sampling								
0–1st	2nd–5th	6th–9th	After 9th	0–1st	2nd–5th	6th–9th	After 9th	
6/23	41/138	53/147	6/17	17/23	97/138	94/147	11/17	0.61
Availability of a main building for animals								
Yes	No			Yes	No			
104/318	2/7			214/318	5/7			0.82
Availability of a designated building for lambs								
Yes	No			Yes	No			
74/243	32/82			169/243	50/82			0.15
Availability of a designated lambing area								
Yes	No			Yes	No			
61/173	45/152			112/173	107/152			0.28
Availability of a lavatory								
Yes	No			Yes	No			
46/143	60/182			97/143	122/182			0.88
Material of the floor of the barn								
Soil	Other			Soil	Other			
96/292	10/33			196/292	23/33			0.76
Availability of straw bedding								
Yes	No			Yes	No			
85/268	21/57			183/268	36/57			0.45
Annual frequency of removal / clean-up of the straw bedding								
1 – 2	> 2			1 – 2	> 2			
48/166	37/102			118/166	65/102			0.21
Grazing practiced								
Yes	No			Yes	No			
97/281	9/44			184/281	35/44			0.06

Grazing land available to animals								
0 – 0.5 acre per animals	> 0.5 acre per animal	0 – 0.5 acre per animals	> 0.5 acre per animal					
39/118	67/207	79/118	140/207					0.90
No. of female animals in the farm								
0 – 165	166 – 330	331 – 500	> 500	0 – 165	166 – 330	331 – 500	> 500	
36/87	37/121	19/66	14/51	51/87	84/121	47/66	37/51	0.23
Breed of ewes								
Cross-breeds	Greek breeds	Imported breeds	Cross-breeds	Greek breeds	Imported breeds			
14/43	49/143	43/139	29/43	94/143	96/139			0.84
Month of the start of the lambing season								
All year	Autumn	Winter	Spring-Summer	All year	Autumn	Winter	Spring-Summer	
4/18	83/245	17/55	2/7	14/18	162/245	38/55	5/7	0.76
Collaboration with a veterinarian								
Yes	No	Yes	No					
94/277	12/48	183/277	36/48					0.22
Use of laboratory diagnostic examinations in faecal samples preventively								
Yes	No	Yes	No					
11/34	95/291	23/34	196/291					0.97
Means of calculating live bodyweight for the administration of pharmaceutical products								
Weighing	Estimation	Weighing	Estimation					
19/73	87/252	54/73	165/252					0.17
Routine overdosing (compared to dose prescribed) of pharmaceuticals								
Yes	No	Yes	No					
21/61	85/264	40/61	179/264					0.74
Total visits made annually by veterinarians to the farm during the preceding season								
0 – 4	5 – 7	> 7	0 – 4	5 – 7	> 7			
47/139	27/86	32/100	92/139	59/86	68/100			0.92
Application of reproductive control practices in the farm								
Yes	No	Yes	No					
39/101	67/224	62/101	157/224					0.12
Newborn care and specific monitoring								
Yes	No	Yes	No					
96/293	10/32	197/293	22/32					0.86
Lamb fostering to female animals other than their dams								
Yes	No	Yes	No					
73/221	33/104	148/221	71/104					0.82

Administration of a lamb-specific diet						
Yes	No	Yes	No			
75/226	31/99	151/226	68/99			0.74
Age of lamb removal from their dams						
< 45 days	45 – 60 days	> 60 days	< 45 days	45 – 60 days	> 60 days	
35/119	61/170	10/36	84/119	109/170	26/36	0.41
Seasonal transfer of animals to other site						
Yes	No	Yes	No			
13/49	93/276	36/49	183/276			0.32
Manure management						
Spread in the fields	Removed otherwise	Spread in the fields	Removed otherwise			
105/320	1/5	215/320	4/5			0.54
Annual frequency of systemic disinfections in the farm						
0 – 9	> 9	0 – 9	> 9			
93/293	13/32	200/293	19/32			0.31
Shearing of animals						
Yes	No	Yes	No			
105/319	1/6	214/319	5/6			0.40
Provision of finished feed (concentrate) to animals throughout the year						
Yes	No	Yes	No			
99/304	7/21	205/304	14/21			0.94
Provision of finished feed (concentrate) to young animals						
Yes	No	Yes	No			
83/255	23/70	172/255	47/70			0.96

Table S9. Associations of presence of *Giardia* in faecal samples from 119 goat herds in Greece, as found in univariable analysis.

Number of farms								p
**Farms with presence of *Giardia* in faecal samples (n = 40)**				**Farms with no presence of *Giardia* in faecal samples (n = 79)**				
Management system applied in the farm								
Intensive	Semi-intensive	Semi-extensive	Extensive	Intensive	Semi-intensive	Semi-extensive	Extensive	
3/9	10/29	20/61	7/20	6/9	19/29	41/61	13/20	0.99
Years at the present location								
0 – 2	3 – 5	> 5		0 – 2	3 – 5	> 5		
0/1	5/15	35/103		1/1	10/15	68/103		0.77
Month into the lactation period at sampling								
0–1st	2nd–5th	6th–9th	After 9th	0–1st	2nd–5th	6th–9th	After 9th	
3/8	19/60	15/43	3/8	5/8	41/60	28/43	5/8	0.97
Availability of a main building for animals								
Yes	No			Yes	No			
38/117	2/2			79/117	0/2			0.045
Availability of a designated building for kids								
Yes	No			Yes	No			
29/86	11/33			57/86	22/33			0.89
Availability of a designated kidding area								
Yes	No			Yes	No			
22/58	18/61			36/58	43/61			0.33
Availability of a lavatory								
Yes	No			Yes	No			
15/38	25/81			23/38	56/81			0.35
Material of the floor of the barn								
Soil	Other			Soil	Other			
36/105	4/14			69/105	10/14			0.67
Availability of straw bedding								
Yes	No			Yes	No			
26/76	14/43			50/76	29/43			0.85
Annual frequency of removal / clean-up of the straw bedding								
1 – 2	> 2			1 – 2	> 2			
16/42	10/34			26/44	24/34			0.43
Grazing practiced								
Yes	No			Yes	No			
38/113	2/6			75/113	4/6			0.99

Grazing land available to animals								
0 – 0.5 acre per animals	> 0.5 acre per animal	0 – 0.5 acre per animals	> 0.5 acre per animal					
5/17	35/102	12/17	67/102					0.69
No. of female animals in the farm								
0 – 165	166 – 330	331 – 500	> 500	0 – 165	166 – 330	331 – 500	> 500	
21/57	7/36	7/13	5/13	36/57	29/36	6/13	8/13	0.11
Breed of does								
Cross-breeds	Greek breeds	Imported breeds	Cross-breeds	Greek breeds	Imported breeds			
6/18	17/56	17/45	12/18	39/56	28/45			0.73
Month of the start of the kidding season								
All year	Autumn	Winter	Spring-Summer	All year	Autumn	Winter	Spring-Summer	
3/8	20/63	16/44	1/4	5/8	43/63	28/44	3/4	0.93
Collaboration with a veterinarian								
Yes	No	Yes	No					
35/101	5/18	66/101	13/18					0.57
Use of laboratory diagnostic examinations in faecal samples preventively								
Yes	No	Yes	No					
6/16	34/103	10/16	69/103					0.72
Means of calculating live bodyweight for the administration of pharmaceutical products								
Weighing	Estimation	Weighing	Estimation					
11/27	29/92	16/27	63/92					0.37
Routine overdosing (compared to dose prescribed) of pharmaceuticals								
Yes	No	Yes	No					
11/26	29/93	15/26	64/93					0.29
Total visits made annually by veterinarians to the farm during the preceding season								
0 – 4	5 – 7	> 7	0 – 4	5 – 7	> 7			
17/51	9/25	14/43	34/51	16/25	29/43			0.96
Application of reproductive control practices in the farm								
Yes	No	Yes	No					
8/17	32/102	9/17	70/102					0.20
Newborn care and specific monitoring								
Yes	No	Yes	No					
37/109	3/10	72/109	7/10					0.80
Kid fostering to female animals other than their dams								
Yes	No	Yes	No					
31/86	9/33	55/86	24/137					0.36

Administration of a kid- specific diet						
Yes	No		Yes	No		
27/79	13/40		52/79	27/40		0.85
Age of kid removal from their dams						
< 45 days	45 – 60 days	> 60 days	< 45 days	45 – 60 days	> 60 days	
8/26	13/44	19/49	18/26	31/44	30/49	0.60
Seasonal transfer of animals to other site						
Yes	No		Yes	No		
8/28	32/91		20/28	59/91		0.52
Manure management						
Spread in the fields	Removed otherwise		Spread in the fields	Removed otherwise		
37/109	3/10		72/109	7/10		0.80
Annual frequency of systemic disinfections in the farm						
0 – 9	> 9		0 – 9	> 9		
36/100	4/19		64/100	15/19		0.21
Shearing of animals						
Yes	No		Yes	No		
35/102	5/17		67/12	12/17		0.69
Provision of finished feed (concentrate) to animals throughout the year						
Yes	No		Yes	No		
34/103	6/16		69/103	10/16		0.72
Provision of finished feed (concentrate) to young animals						
Yes	No		Yes	No		
27/79	13/40		52/79	27/40		0.85

Table S10. Associations of presence of *Cryptosporidium* in faecal samples from 325 sheep flocks in Greece, as found in univariable analysis.

**Number of farms**								***p***
**Farms with presence of *Giardia* in faecal samples (*n* = 23)**				**Farms with no presence of *Giardia* in faecal samples (*n* = 302)**				
Management system applied in the farm								
Intensive	Semi-intensive	Semi-extensive	Extensive	Intensive	Semi-intensive	Semi-extensive	Extensive	
14/43	8/151	1/107	0/24	29/43	143/151	106/107	24/24	< 0.0001
Years at the present location								
0 – 2	3 – 5	> 5		0 – 2	3 – 5	> 5		
2/5	2/40	19/280		3/5	38/40	261/280		0.014
Month into the lactation period at sampling								
0–1st	2nd–5th	6th–9th	After 9th	0–1st	2nd–5th	6th–9th	After 9th	
2/23	7/138	12/147	2/17	21/23	131/138	135/147	15/17	0.62
Availability of a main building for animals								
Yes	No			Yes	No			
23/318	0/7			295/318	7/7			0.46
Availability of a designated building for lambs								
Yes	No			Yes	No			
21/243	2/82			222/243	80/82			0.06
Availability of a designated lambing area								
Yes	No			Yes	No			
12/173	11/152			161/173	141/152			0.92
Availability of a lavatory								
Yes	No			Yes	No			
14/143	9/182			129/143	173/182			0.09
Material of the floor of the barn								
Soil	Other			Soil	Other			
19/292	4/33			273/292	29/33			0.23
Availability of straw bedding								
Yes	No			Yes	No			
21/268	2/57			247/268	55/57			0.25
Annual frequency of removal / clean-up of the straw bedding								
1 – 2	> 2			1 – 2	> 2			
10/164	11/102			156/164	91/102			0.16
Grazing practiced								
Yes	No			Yes	No			
10/281	13/44			271/281	31/44			< 0.0001

Grazing land available to animals								
0 – 0.5 acre per animals	> 0.5 acre per animal	0 – 0.5 acre per animals	> 0.5 acre per animal					
19/118	4/207	99/118	203/207					< 0.0001
No. of female animals in the farm								
0 – 165	166 – 330	331 – 500	> 500	0 – 165	166 – 330	331 – 500	> 500	
4/87	6/121	7/66	6/51	83/87	115/121	59/66	45/51	0.21
Breed of ewes								
Cross-breeds	Greek breeds	Imported breeds	Cross-breeds	Greek breeds	Imported breeds			
0/43	8/143	15/139	43/43	135/143	144/139			0.07
Month of the start of the lambing season								
All year	Autumn	Winter	Spring-Summer	All year	Autumn	Winter	Spring-Summer	
4/18	11/245	6/55	2/7	14/18	234/245	49/55	5/7	0.002
Collaboration with a veterinarian								
Yes	No	Yes	No					
21/277	2/48	256/277	46/48					0.39
Use of laboratory diagnostic examinations in faecal samples preventively								
Yes	No	Yes	No					
4/34	19/291	30/34	272/291					0.26
Means of calculating live bodyweight for the administration of pharmaceutical products								
Weighing	Estimation	Weighing	Estimation					
3/73	20/252	70/73	232/252					0.26
Routine overdosing (compared to dose prescribed) of pharmaceuticals								
Yes	No	Yes	No					
7/61	16/264	54/61	248/264					0.14
Total visits made annually by veterinarians to the farm during the preceding season								
0 – 4	5 – 7	> 7	0 – 4	5 – 7	> 7			
11/139	4/86	8/100	128/139	82/86	92/100			0.59
Application of reproductive control practices in the farm								
Yes	No	Yes	No					
9/101	14/224	92/101	210/224					0.39
Newborn care and specific monitoring								
Yes	No	Yes	No					
22/293	1/32	271/293	31/32					0.36
Lamb fostering to female animals other than their dams								
Yes	No	Yes	No					
11/221	12/104	210/221	92/104					0.031

Administration of a lamb-specific diet						
Yes	No		Yes	No		
15/226	8/99		211/226	91/99		0.64
Age of lamb removal from their dams						
< 45 days	45 – 60 days	> 60 days	< 45 days	45 – 60 days	> 60 days	
9/119	12/170	2/36	110/119	158/170	34/36	0.92
Seasonal transfer of animals to other site						
Yes	No		Yes	No		
2/49	21/276		47/49	255/276		0.37
Manure management						
Spread in the fields	Removed otherwise		Spread in the fields	Removed otherwise		
23/320	0/5		297/320	5/5		0.53
Annual frequency of systemic disinfections in the farm						
0 – 9	> 9		0 – 9	> 9		
18/293	5/32		275/293	27/32		0.047
Shearing of animals						
Yes	No		Yes	No		
23/319	0/6		296/319	6/6		0.50
Provision of finished feed (concentrate) to animals throughout the year						
Yes	No		Yes	No		
23/304	0/21		281/304	21/21		0.19
Provision of finished feed (concentrate) to young animals						
Yes	No		Yes	No		
18/255	5/70		237/255	65/70		0.98

Table S11. Associations of presence of *Cryptosporidium* in faecal samples from 119 goat herds in Greece, as found in univariable analysis.

**Number of farms**								p
**Farms with presence of *Cryptosporidium* in faecal samples (n = 13)**				**Farms with no presence of *Cryptosporidium* in faecal samples (n = 106)**				
Management system applied in the farm								
Intensive	Semi-intensive	Semi-extensive	Extensive	Intensive	Semi-intensive	Semi-extensive	Extensive	
6/9	7/29	0/61	0/20	3/9	22/29	61/61	20/20	< 0.0001
Years at the present location								
0 – 2	3 – 5	> 5		0 – 2	3 – 5	> 5		
0/1	2/15	11/103		1/1	13/15	92/103		0.90
Month into the lactation period at sampling								
0–1st	2nd–5th	6th–9th	After 9th	0–1st	2nd–5th	6th–9th	After 9th	
1/8	5/60	5/43	2/8	7/8	55/60	38/43	6/8	0.55
Availability of a main building for animals								
Yes	No			Yes	No			
13/117	0/2			104/117	2/2			0.62
Availability of a designated building for lambs / kids								
Yes	No			Yes	No			
10/86	3/33			83/86	30/33			0.79
Availability of a dedicated lambing / kidding area								
Yes	No			Yes	No			
5/58	8/61			53/58	53/61			0.43
Availability of a lavatory								
Yes	No			Yes	No			
6/38	7/81			32/38	74/81			0.24
Material of the floor of the barn								
Soil	Other			Soil	Other			
12/105	1/14			93/105	13/14			0.63
Availability of straw bedding								
Yes	No			Yes	No			
11/76	2/43			65/76	41/43			0.10
Annual frequency of removal / clean-up of the straw bedding								
1 – 2	> 2			1 – 2	> 2			
3/42	8/34			39/42	26/34			0.044
Grazing practiced								
Yes	No			Yes	No			
9/113	4/6			104/113	2/6			< 0.0001

Grazing land available to animals								
0 – 0.5 acre per animals	> 0.5 acre per animal	0 – 0.5 acre per animals	> 0.5 acre per animal					
7/17	6/102	10/17	96/102					< 0.0001
No. of female animals in the farm								
0 – 165	166 – 330	331 – 500	> 500	0 – 165	166 – 330	331 – 500	> 500	
4/57	3/36	5/13	1/13	53/57	33/36	8/13	12/13	0.010
Breed of ewes / does								
Cross-breeds	Greek breeds	Imported breeds	Cross-breeds	Greek breeds	Imported breeds			
1/18	2/56	10/45	17/18	54/56	35/45			0.008
Month of the start of the lambing / kidding season								
All year	Autumn	Winter	Spring-Summer	All year	Autumn	Winter	Spring-Summer	
4/8	3/62	6/44	0/5	4/8	59/62	38/44	5/5	0.001
Collaboration with a veterinarian								
Yes	No	Yes	No					
11/101	2/18	90/101	16/18					0.98
Use of laboratory diagnostic examinations in faecal samples preventively								
Yes	No	Yes	No					
4/16	9/103	12/16	94/103					0.05
Means of calculating live bodyweight for the administration of pharmaceutical products								
Weighing	Estimation	Weighing	Estimation					
4/87	9/32	83/87	23/32					0.0003
Routine overdosing (compared to dose prescribed) of pharmaceuticals								
Yes	No	Yes	No					
2/26	11/93	24/26	82/93					0.55
Total visits made annually by veterinarians to the farm during the preceding season								
0 – 4	5 – 7	> 7	0 – 4	5 – 7	> 7			
6/51	3/25	4/43	45/51	22/25	39/43			0.91
Application of reproductive control practices in the farm								
Yes	No	Yes	No					
4/17	9/102	13/17	93/102					0.07
Newborn care and specific monitoring								
Yes	No	Yes	No					
13/109	0/10	96/109	10/10					0.25
Kid fostering to female animals other than their dams								
Yes	No	Yes	No					
6/86	7/33	80/86	26/33					0.026

Administration of a kid-specific diet						
Yes	No		Yes	No		
9/88	4/31		79/88	27/31		0.68
Age of kid removal from their dams						
< 45 days	45 – 60 days	> 60 days	< 45 days	45 – 60 days	> 60 days	
5/26	5/44	3/49	21/26	39/44	46/49	0.22
Seasonal transfer of animals to other site						
Yes	No		Yes	No		
2/28	11/91		26/28	80/91		0.46
Manure management						
Spread in the fields	Removed otherwise		Spread in the fields	Removed otherwise		
11/109	2/10		98/109	8/10		0.34
Annual frequency of systemic disinfections in the farm						
0 – 9	> 9		0 – 9	> 9		
10/100	3/19		90/100	16/19		0.46
Shearing of animals						
Yes	No		Yes	No		
10/102	3/17		92/102	14/17		0.34
Provision of finished feed (concentrate) to animals throughout the year						
Yes	No		Yes	No		
13/103	0/16		90/103	16/16		0.13
Provision of finished feed (concentrate) to young animals						
Yes	No		Yes	No		
9/79	4/40		70/79	36/40		0.82
